# Supplementary material for: Getting over the hurdles to save lives: Incorporating perceived barriers into theory of planned behaviour (TPB) model to predict stated intention among Hong Kong trained laymen
Source: PLoS One. 2026 Jun 4;21(6):e0350392. doi: 10.1371/journal.pone.0350392 (PMC13235877; doi:10.1371/journal.pone.0350392)
Supplement: S1 Table — (DOCX) [file pone.0350392.s001.docx]

**S1 Table. Additional internal consistency indicators (N=678)**

|  | Item number | Cronbach's α | AVE | CR |
| --- | --- | --- | --- | --- |
| **TPB model constructs** |  |  |  |  |
| BLS Intention | 2 | 0.833 | 0.714 | 0.833 |
| Attitude | 4 | 0.765 | 0.533 | 0.819 |
| Subjective norms | 5 | 0.916 | 0.687 | 0.915 |
| Perceived behavioural control | 4 | 0.937 | 0.788 | 0.937 |
| **Lumped barriers model construct** |  |  |  |  |
| Perceived barriers | 5 | 0.689 | 0.310 | 0.646 |
| **Separate barriers model construct** |  |  |  |  |
| Performance-related barriers | 2 | 0.759 | 0.620 | 0.765 |
| Cultural barriers | 2 | 0.710 | 0.559 | 0.716 |

Note. Acceptable internal consistency was determined by (i) Composite reliability (CR) ≥ 0.70, and (ii) average variance extracted (AVE) ≥ 0.50.
