# Supplementary material for: Getting over the hurdles to save lives: Incorporating perceived barriers into theory of planned behaviour (TPB) model to predict stated intention among Hong Kong trained laymen
Source: PLoS One. 2026 Jun 4;21(6):e0350392. doi: 10.1371/journal.pone.0350392 (PMC13235877; doi:10.1371/journal.pone.0350392)
Supplement: S2 Table — (DOCX) [file pone.0350392.s002.docx]

**S2 Table. Heterotrait-Monotrait (HTMT) ratios (N=678)**

|  | **1** | **2** | **3** | **4** | **5** | **6** | **7** |
| --- | --- | --- | --- | --- | --- | --- | --- |
| 1. BLS Intention | - |  |  |  |  |  |  |
| 2. Attitude | 0.558 | - |  |  |  |  |  |
| 3. Subjective norms | 0.652 | 0.567 | - |  |  |  |  |
| 4. Perceived behavioural control | 0.758 | 0.497 | 0.674 | - |  |  |  |
| 5. Perceived barriers | 0.492 | 0.473 | 0.461 | 0.599 | - |  |  |
| 6. Performance-related barriers | 0.596 | 0.355 | 0.490 | 0.734 | NA | - |  |
| 7. Cultural barriers | 0.217 | 0.444 | 0.293 | 0.286 | NA | 0.416 | - |

Note. Acceptable discriminant validity was determined by a HTMT ratio <0.85.
