# Supplementary material for: Getting over the hurdles to save lives: Incorporating perceived barriers into theory of planned behaviour (TPB) model to predict stated intention among Hong Kong trained laymen
Source: PLoS One. 2026 Jun 4;21(6):e0350392. doi: 10.1371/journal.pone.0350392 (PMC13235877; doi:10.1371/journal.pone.0350392)
Supplement: Attachment — Submitted filename: Responses to Reviewers.docx [file pone.0350392.s005.docx]

**Responses to reviewers**

RE: Getting Over the Hurdles to Save Lives: Incorporating Perceived Barriers Into Theory Of Planned Behaviour (TPB) Model To Predict Stated Intention Among Hong Kong Trained Laymen (PONE-D-25-57612)

Reviewer #1:

1) Theoretical and Applied Contribution

The manuscript represents a relevant contribution by integrating perceived barriers within the Theory of Planned Behavior (TPB) and empirically demonstrating how these barriers influence the intention to act through the model’s three core determinants: attitude, subjective norms (SN), and perceived behavioral control (PBC).
The strength of the study lies in the fact that it does not simply describe obstacles but proposes an explanatory and applicable model that helps to understand how and why certain barriers reduce the willingness to intervene. Theoretically, it expands the TPB to contexts where the decision to act must be made in seconds (such as in out-of-hospital cardiac arrests), while practically, it translates the findings into specific educational recommendations: reinforcing normative messages, self-efficacy, social modeling, and legal reassurance.
This connection between theory and application makes the study a useful tool for CPR/AED trainers, health organizations, and curriculum designers involved in prevention and emergency response programs.

*Responses: Thank you for your positive comments.*

2) Analytical Rigor

The analysis is consistent with the objectives and demonstrates a solid statistical structure. First, the exploratory factor analysis (EFA) helped to define the internal structure of the perceived barriers. Then, the structural equation modeling (SEM) compared two approaches: a unified (“lumped”) model and a divided (“split”) model with three subfactors (cultural, performance, and infection-related barriers), showing a better fit for the split model.
This indicates that barriers do not operate through the same psychological pathways and supports differentiating them for educational and diagnostic purposes. The reporting of internal consistency (Cronbach’s), correlations, and test–retest adds further evidence of reliability and temporal stability. Although additional details about normality testing or estimators would be helpful, the analysis meets international standards for validation and structural modeling in behavioral sciences.

*Responses: Thank you for your positive comments. We have fine-tuned Model 1 by adding covariates between the TPB construct error terms, reflecting the shared variance not explained by the predictors. For Model 2, we have revised the analysis in line with Reviewer 2’s comments to isolate concerns about infection and to conduct a separate multigroup analysis to further examine its potential moderating role (L211-228). The revised Model 2 has a satisfactory model fit and resolved problematic paths. The results differ slightly from the original Model 2, while the structural pattern remains stable: performance-related barriers are strongly associated with PBC, and cultural barriers are strongly associated with attitude but not with PBC (L211-217). We believe that this modification would enhance the robustness of the SEM and provide a unique opportunity to evaluate the effect of concerns about infection.*

3) Public Health Relevance
The study identifies three types of barriers, cultural, performance-related, and infection-related, with direct implications for intention-focused education. The findings can be translated as follows:
Cultural barriers: Address deep-rooted beliefs (fear, superstition, gender roles) and strengthen social norms.
Performance barriers: Reinforce self-efficacy, guided practice, and simulations.
Infection barriers: Clarify safety protocols and promote compression-only CPR.
The research goes beyond the local context, as the mechanisms it explains allow for adaptation to other populations facing similar cultural or legal barriers. It is a valuable contribution to public health education and civic engagement in emergency response.

*Responses: Thank you again for your positive comments and for highlighting the translation of our findings. We have further strengthened the discussion in highlighting the public health relevance of each barrier in L278-284.*

Points for Improvement
1) Sampling Details
The text alternates between convenience and snowball sampling. It would be useful to unify the terminology and clarify the recruitment process (platforms used, filters applied, response rate, and sample characteristics).

*Responses: We have revised the use of convenience sampling throughout the text. Platforms used and sample characteristics have been reported in L101 and 145-150, respectively.*

Conclusion
In summary, the manuscript is technically sound, theoretically well-grounded, and has clear applied potential. It offers both scientific rigor and practical relevance, positioning itself as a useful reference for developing intention-focused training in bystander resuscitation and other public health interventions.

*Responses: We sincerely thank you for your positive and constructive comments, which have helped enhance our manuscript particular the discussion and public health implications. Thank you.*

Reviewer #2:

Dear Author,
This manuscript addresses an important public health topic and applies the Theory of Planned Behavior (TPB) framework to examine BLS intention among lay individuals in Hong Kong. The sample size is adequate, and the use of structural equation modeling (SEM) is appropriate in principle. However, several methodological and statistical concerns must be clarified before the findings can be interpreted with confidence.

*Overall responses: Thank you so much for your constructive comments, which help us revise the manuscript. We have substantially revised the data analysis and the discussion of limitations in response to your comments. You may refer to the point-by-point responses below. Alongside the substantial revision in data analysis, we would like to* ***reposition the focus of this manuscript to examine the structural relationships among variables using SEM, while reducing the emphasis on scale validation****. Therefore, we also revised the title to better fit the revised manuscript. We believe that this fine-tuning of the study concept would provide a more focused discussion of the incorporation of perceived barriers into predicting BLS intention among trained laypeople, adding a concise psychological perspective to future BLS training strategies. Thank you again for your time and effort.*

1. Several reported standardized path coefficients exceed the theoretical bounds of ±1 (e.g., β = -1.41; β = -1.65; β = 1.24). Standardized regression coefficients should fall between -1 and +1. These values raise concerns regarding model misspecification, multicollinearity, suppression effects, or possible reporting errors (standardized vs. unstandardized estimates). Please clarify and provide appropriate diagnostics.

*Responses: Thank you for the constructive comments. We acknowledged that the paths related to the concerns about infection appeared problematic in the SEM. After consulting a psychology expert, our team conducted the analysis again, thoroughly examining the role of concerns about infection as a potential moderator rather than an isolated barrier. This adjustment is justified by the unique data-collection period during the prolonged 5th wave of the local COVID-19 outbreak spanning 2021-2022, when local community members may have had varying levels of fear of contracting an infectious disease (Chair et. al., 2023).*

*We hypothesized that levels of concern about infection moderate the structural relationship of the proposed model. To test this hypothesis, a multigroup analysis was conducted. The methods and results have been detailed in L134-143 and L222-228, respectively. We believe that by understanding the effect of concerns about infection on the relationship between perceived barriers, TPB constructs and BLS intention during this special period of prolonged COVID-19 wave could offer insights into future lay people BLS training in this “new normal” post-pandemic era.*

*Reference: Chair SY, Chien WT, Liu T, Lam L, Cross W, Banik B, et al. Psychological Distress, Fear and Coping Strategies among Hong Kong People During the COVID-19 Pandemic. Current Psychology. 2023;42(3):2538–57. doi: 10.1007/s12144-021-02338-7*

2. Bivariate analyses indicate that concerns about infection are negatively correlated with intention and TPB determinants. However, the SEM results show strong positive structural paths. This discrepancy requires statistical and theoretical explanation.

*Responses: Please see the response in the Q1 for details on revision of data analysis. This concern has been resolved in the revised analysis.*

3. Given the high inter-correlations among constructs (e.g., r = 0.84), Cronbach’s alpha alone is insufficient. Please report additional psychometric indicators such as AVE, Composite Reliability, and discriminant validity metrics (e.g., Fornell-Larcker criterion or HTMT ratios).

*Responses: We acknowledged that some constructs have shown strong correlations, particularly among the barrier variables. An exceptionally high correlation has been demonstrated between Perceived Barriers (PB) and Performance-related barriers (r = 0.84), as well as relatively high correlations between PB and Concerns about Infection (r = 0.61) and PB and Cultural barriers (r = 0.69). We would like to reassure you that these constructs are not included in the same model; rather, they represent separate, lumped models that were tested.*

*Another high correlation was noted between Perceived Behavioural Control and Performance-related barriers. We acknowledged this strong negative correlation as conceptually there are highly relevant (i.e., having great psychological barriers in performing well 🡪 Feel that they are not confident to do so).*

*Despite a reduction in emphasis on scale validation, we would like to provide additional psychometric indicators in the supplementary tables 1 & 2 as stated in L168-169. Thank you.*

4. The study employed convenience sampling via social media platforms. While acknowledged, the implications of self-selection bias, unknown response rate, and digital sampling limitations require deeper discussion.

*Responses: Thank you for the suggestion. Self-selection bias, unknown response rate, and digital sampling limitations were furthered discussed in L319-329.*

5. The proposed TPB pathways imply directionality; however, the cross-sectional design precludes causal inference. This limitation should be more explicitly acknowledged.

*Responses: The limitation of the cross-sectional design and its potential misinterpretation have been further discussed in L337-340. Readers are reminded to interpret the findings cautiously and not to misconstrue them as indicative of temporal or causal effects.*

6. The transition from 1449 respondents to 678 analyzed participants is insufficiently described. Please provide a detailed breakdown (preferably with a flow diagram).

*Responses: Thank you for the suggestion. Figure 1 has been added to illustrate the detailed steps of extracting the responses.*

7. The reported monthly income categories (e.g., <$15,000) appear implausible and may reflect a unit or currency misclassification. Please clarify.

*Responses: Revised to HK$ to specify the currency (L149-150), thank you.*

8. The statement claiming this is the first study examining BLS intention among “lay rescuers in HK” appears overly broad. The sample consists specifically of self-reported trained lay rescuers recruited via social media. The wording should be revised to avoid overgeneralization.

*Responses: This statement has been revised to highlight that no previous study has been conducted in the local context (i.e., Hong Kong), rather than the specific lay rescuer population (L235-226). We further acknowledged that the sample may not be representative of all lay rescuers in HK (L319-325).*

9. The current limitations section does not adequately address:
Model instability concerns
Construct overlap
Cross-sectional design constraints
Potential common method bias
A more comprehensive limitations discussion is necessary.

*Responses: Thank you for the highlighting the area for improvement. The limitations section has been substantially revised (L319-340), including potential common method bias, such as volunteer bias and non-response bias. Discussion of construct overlap, model instability concerns, and Cross-sectional design constraints has been presented, along with suggestions for future study directions.*
